# Comprehensive analysis of PD-L1 expression in glioblastoma multiforme

**DOI:** 10.18632/oncotarget.15031

**Published:** 2017-02-02

**Authors:** Dieter Henrik Heiland, Gerrit Haaker, Daniel Delev, Bianca Mercas, Waseem Masalha, Sabrina Heynckes, Annette Gäbelein, Dietmar Pfeifer, Maria Stella Carro, Astrid Weyerbrock, Marco Prinz, Oliver Schnell

**Affiliations:** ^1^ Department of Neurosurgery, Medical Center - University of Freiburg, Baden-Württemberg, Germany; ^2^ Department of Hematology, Oncology and Stem Cell Transplantation, Medical Center - University of Freiburg, Baden-Württemberg, Germany; ^3^ Institute of Neuropathology, Medical Center - University of Freiburg, Baden-Württemberg, Germany; ^4^ Faculty of Medicine, University of Freiburg, Baden-Württemberg, Germany; ^5^ BIOSS Centre for Biological Signalling Studies, University of Freiburg, Baden-Württemberg, Germany

**Keywords:** glioblastoma multiforme, PD-L1, WGCNA, integrative analysis, immunotherapy

## Abstract

Glioblastoma multiforme are highly malignant brain tumours with frequent genetic and epigenetic alterations. The poor clinical outcome of these tumours necessitates the development of new treatment options. Immunotherapies for glioblastoma multiforme including PD1/PD-L1 inhibition are currently tested in ongoing clinical trials. The purpose of this study was to investigate the molecular background of *PD-L1* expression in glioblastoma multiforme and to find associated pathway activation and genetic alterations. We show that *PD-L1* is up-regulated in *IDH1/2* wildtype glioblastoma multiforme compared to lower-grade gliomas. In addition, a strong association of *PD-L1* with the mesenchymal expression subgroup was observed. Consistent with that, *NF1* mutation and corresponding activation of the MAPK pathway was strongly connected to *PD-L1* expression. Our findings may explain different response to PD-L1 inhibition of patients in ongoing trials and may help to select patients that may profit of immunotherapy in the future.

## INTRODUCTION

Glioblastoma multiforme (WHO grade IV) is the most common type of brain tumours, which is characterised by poor clinical outcome and short survival time, rarely longer than 14 months [1]. Extensive efforts have been made to develop new treatment strategies during the last decades, without improving the poor clinical course of glioblastoma multiforme [2, 3]. Until now, the “gold standard” in glioblastoma multiforme therapy remains surgery plus adjuvant combined chemoradiotherapy introduced by Stupp et al., 2005 [4].

### Clinical background

Glioblastoma multiforme contains a high frequency of genetic and epigenetic alterations with numerous potentially produced neoantigens [5], which are recognized by the immune system and support T-cell based immune response [5]. So far, new treatment strategies are investigated for clinical testing. Those treatments target the programmed death ligand 1 (*PD-L1*) or the programmed death ligand protein 1 (*PD-1*) receptor and attempt to re-activate the immune system. Currently, PD-1/PD-L1 therapeutic antibodies are investigated for a large variety of different cancer types. For instance, in small-cell lung carcinoma and melanoma, PD-1 inhibitors were successfully tested in clinical trials and could significantly improve the overall survival [6–8].

### PD-L1 regulation

The programmed death ligand 1 (*PD-L1* or *CD274*) plays a major role in preventing the immune response in many cancer types [6, 8, 9]. This mechanism of immune system escape is described as “tumour immunity” [10–12] and represents as a hallmark of cancer biology. Recent studies have reported a strong occurrence of *PD-L1* in glioblastoma, which is targetable by prospective immunotherapies [13]. The underling regulation mechanism is not well described and rarely explored. Bloch et al. 2013 reported a regulation of PD-L1 by tumour associated macrophages and related Il-10 signalling [14]. Some years ago, Parsa et al., 2006 showed a relationship between loss of *PTEN* and increased expression of *PD-L1* in glioblastoma multiforme [10]. However, the association was not observed in following studies as the study by Berghoff et al., 2015 [13]. Glioblastoma multiforme are tumours with an aggressive local growth pattern, including strong migration, proliferation and invasion of normal brain [15]. Invasive parts of glioblastoma multiforme were associated with massive metabolic alterations within hypoxia and HIF1A up-regulation which lead to an increased expression of PD-L1 [16].

Until now, genetic alterations and specific tumour related pathways that support PD-L1 depended “immune escape” in glioblastoma multiforme are rarely explored.

In other cancer types, *PD-L1* expression and its clinical impact are more frequently investigated. Madore et al., described a strong association between *PD-L1* expression and mutational load in melanoma [17]. In addition, an up-regulation of immune response pathways was found in *PD-L1* positive melanoma [17]. Another study investigated the regulation mechanism of *PD-L1* by activation of PI(3)-kinase and phospho-S6-kinase in breast cancer [18]. Chen et al., described different mechanism of *PD-L1* regulation as PI(3)Kinase and MAPK pathway [19]. Also, an association of the hypoxic pathway and *STAT3* transcriptional regulation was found [16, 20].

The study reported here, combines data from both the *TCGA Data Platform* and local tumour-bank in order to perform an integrative analysis of *PD-L1* expression in glioblastoma multiforme. These results may improve further diagnostics and identify glioblastoma multiforme subgroups that could profit from PD-L1 targeted therapy and may explain different clinical courses of patients in ongoing clinical trials.

## RESULTS

### PD-L1 expression/methylation patterns in glioma subgroups

**Figure 1:**
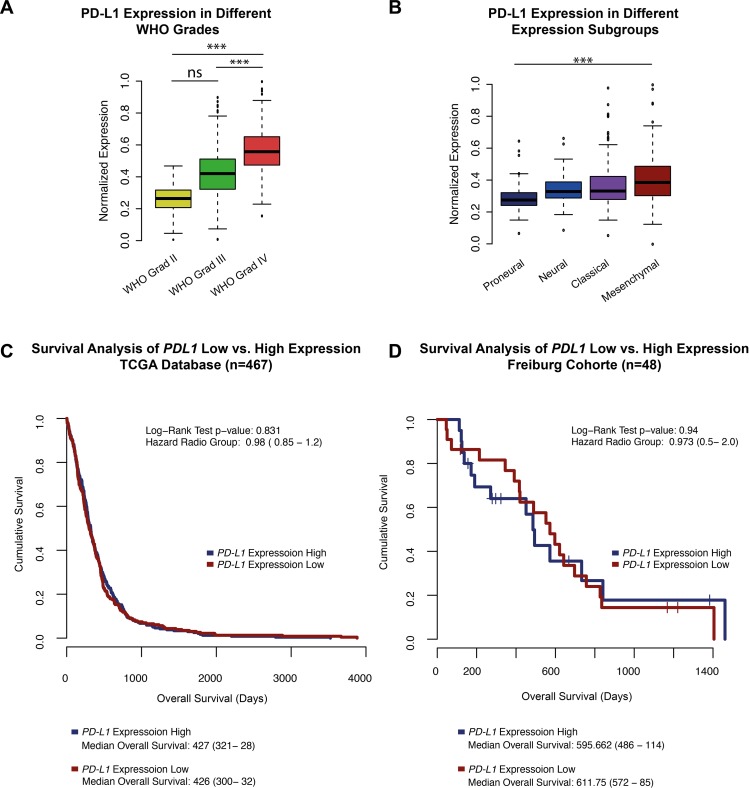
**A**. A boxplot *PD-L1* expression in patients with WHO grad II, III and glioblastoma multiforme (WHO grade IV). **B**. A boxplot of *PD-L1* expression in all expression subclasses define by Verhaak et al., 2010 of high-grade gliomas (glioblastoma multiforme). **C-D**. Survival Analysis of the TCGA database and Freiburg cohort. PD-L1 high vs low was determinate by the mean expression +/− standard deviation. Patients of the Freiburg cohort with outstanding events were censored and marked by a cross. *** p<0.001, ** p=0.01, *p<0.05.

*PD-L1* was differently expressed according to the tumour grade (Figure 1A). WHO grade IV tumours, showed significantly stronger expression compared to lower-grade gliomas (WHOII-WHOIII p>0.05, WHOII/WHOIII-WHOIV p<0.05). Moreover, analysis of PD-L1 expression in different glioblastoma multiforme subgroups (Verhaak et al., 2010 [21]) revealed an increased expression of *PD-L1* in mesenchymal tumour samples (p<0.05) (Figure 1B), whereas the lowest *PD-L1* expression was found in proneural tumours.

Survival analysis of the TCGA patients showed no differences in the overall survival (OS) of patients with high *PD-L1* expression (define as: mean *PD-L1* expression plus standard deviation, mean OS: 427 days) or low *PD-L1* expression (define as: mean *PD-L1* expression minus standard deviation, mean OS: 426 days) with a Hazard ratio (HR) of 0.98 (p>0.05), Figure 1C. Similar results were found in the Freiburg cohort with a mean OS of 595 day in the PD-L1-high group vs. 611 days in patients with low PD-L1 expression (HR 0.94 p>0.05), Figure 1D.

**Figure 2:**
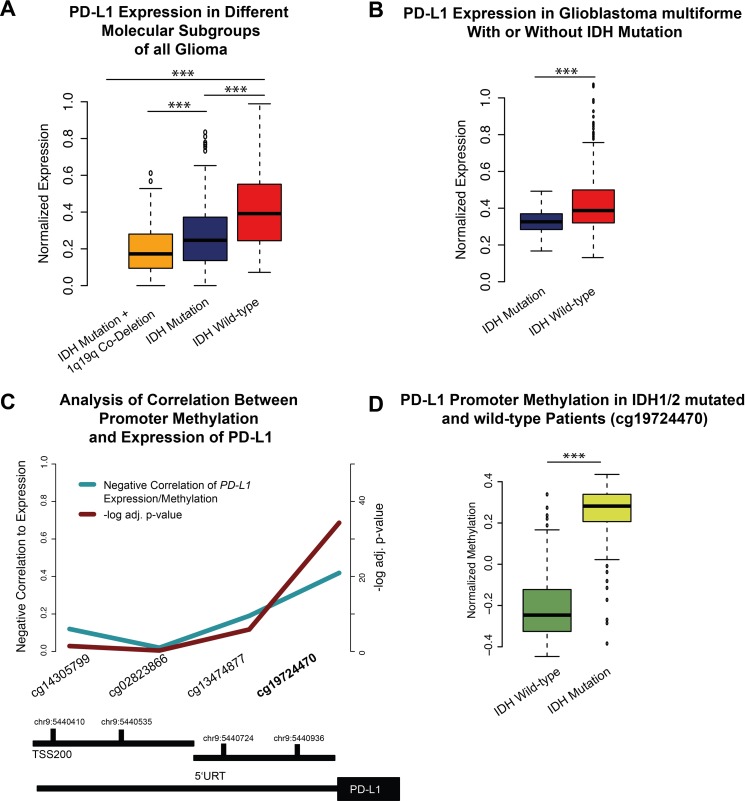
**A**. A boxplot of *PD-L1* expression in lower-grade gliomas and its molecular subgroups. **B**. A boxplot of *PD-L1* expression in IDH mutated and non-mutated glioblastoma multiforme. **D**. A boxplot of *PD-L1* promoter methylation in *IDH1/2* wild-type and mutated patients. **C**. Functional analysis of methylation/expression correlation of different CpG sides of the PD-L1 promoter. The strongest negative correlation was found in the cg19724470. Significant values were corrected by false-discover rate (FDR). (D) This CpG side was used to validate the mean methylation of IDH mutated vs. non-mutated patients. *** p<0.001, ** p=0.01, *p<0.05.

Brad et al., described three distinct molecular subtypes in lower-grade gliomas (WHO II-III) [22]. Analysis of these subgroups (*IDH1/2* mutation, *IDH1/2* mutation+1p19q Co-deletion, *IDH1/2* wild-type) revealed a strong *PD-L1* expression in the *IDH1/2* wild-type samples (Figure 2A). The methylom of patients with *IDH1/2* mutation presented enormous epigenetic alterations, described as the hypermethylated phenotype (G-CIMP) [23]. Those alterations may influence the PD-L1 expression by epigenetic silencing. In an additional methylation analysis of the *PD-L1* promoter region, a hypermethylation in *IDH1/2* mutated patients was found (Figure 1C, 1D), possibly explaining the downregulation of *PD-L1* expression in proneural glioblastoma, which are partly G-CIMP (Figure 1D). Similar findings were investigated in glioblastoma multiforme patients with and without IDH-mutation (p<0.01).

### Copy-number-variations (CNV) and mutational analysis

**Figure 3:**
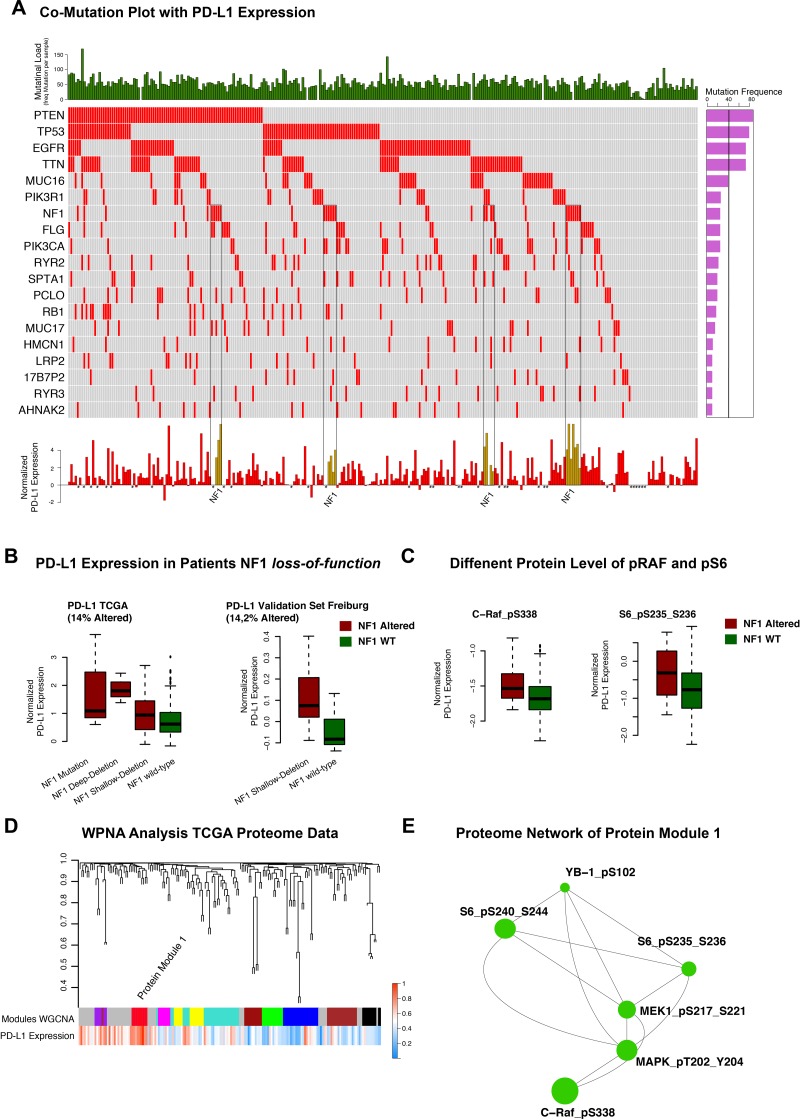
**A**. Co-mutation plot of highly frequent mutations in glioblastoma multiforme. Mutational frequencies were given in a bar plot beside the co-mutation plot. General mutation load as total numbers of mutations in each patient was presented in the top barplot. PD-L1 expression was illustrated in a barplot in the panel below. Patients with missing expression values were marked by *. **B**. Boxplots of PD-L1 expression (TCGA) in NF1 wild-type and mutated/deleted samples were given in the left panel. The validation cohort of Freiburg patients showed similar expression differences (right panel). **C**. Protein-level of p-c-RAF and p-S6 in mutated/deleted and wild- type samples were illustrated. **D**. Weighted Proteome Network Analysis of the proteome data investigated protein module 1 as highly correlated to PD-L1 expression level. **E**. Created network analysis of protein module 1 based on intramodule connectivity, derived from WPNA.

Whole-exome sequencing data of 264 patients derived from the TCGA database were analysed. 19 mutations with high occurrence were selected as shown in Figure 3A. Expression of *PD-L1* was not directly correlated to the total mutation load of each patient (R=0.2 p>0.05). However, after performing a logistic regression analysis, *NF1* mutation was identified as a predictor of increased *PD-L1* expression. Other very frequently occurring mutations like *PTEN* (p>0.05), *EGFR* (p>0.05) or *TP53* (p>0.05) did not correlate with *PD-L1* expression. However, the effect of *NF1* alteration on *PD-L1* expression was not exclusively for *NF1* mutation, also patients with *NF1* deletions showed increased expression of *PD-L1* (p<0.01 for homozygote and heterozygote deletions). These findings could be confirmed in a validation cohort (Freiburg) (p<0.01 heterozygote deletions) (Figure 3B). *IDH 1/2* mutation was found negatively associated with *PD-L1* expression (as described above). This result is in line with the above-described *PD-L1* expression in the *IDH1/2* mutated subgroups.

### Weighted proteome correlation network analysis (WPCNA)

**Figure 4:**
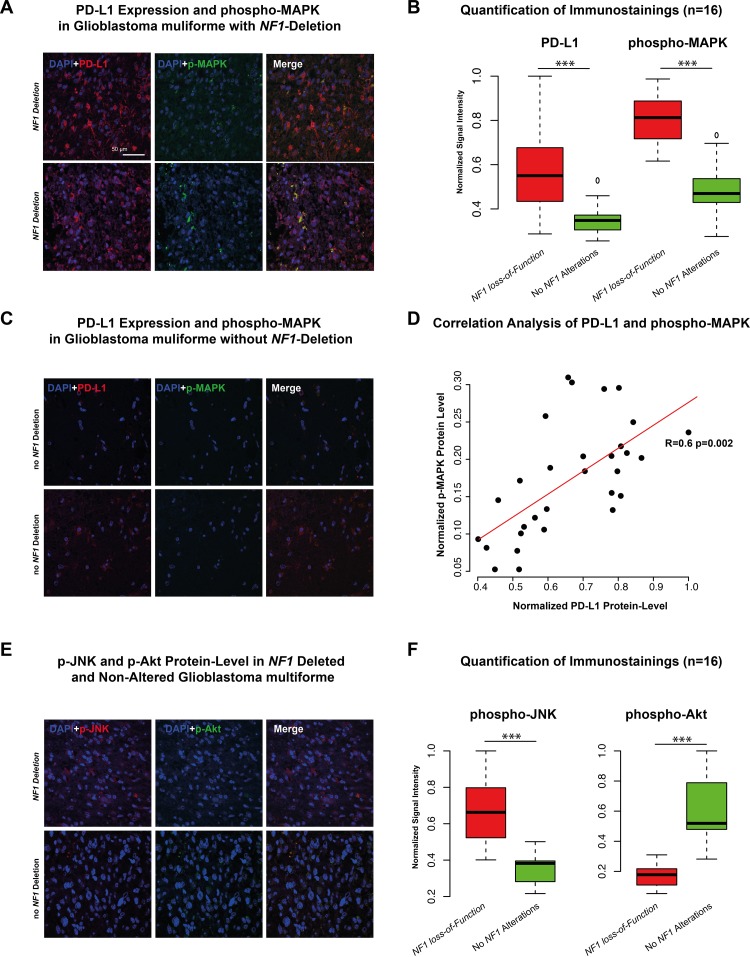
**A-C**. Immunostaining of PD-L1 and phospho-MAPK14 in two patients with NF1 deletion **(A)** and two patient without NF1 alteration **(C)**. Additionally, six independent fields were quantified by mean signal intensity by ImageJ, normalized and illustrated in a boxplot. **D**. Correlation analysis of protein-level of PD-L1 and p-MAPK showed a strong positive correlation (r=0.6 p<0.01). **E-F**. Immunostaining of phospho-JNK and phospho-AKT in a patient with NF1 deletion (upper panel) and one patient without NF1 alteration (lower panel). Additionally, quantification was given in the right boxplot (F). *** p<0.001, ** p=0.01, *p<0.05.

Proteome data of the TCGA database were used as input for a network analysis. The purpose of the analysis was to describe a *PD-L1* regulatory network at protein level and connected proteins were summarised in modules by WPNA (Figure 3D). Next, the modules that showed an association with *PD-L1* expression were analysed by its intramodule-connectivity in correlation to *PD-L1* expression. Protein module 1 (PM1) was identified as highly correlated to *PD-L1* expression (R=0.32 p=2.0E-5). A network was calculated based on the intramodule connectivity of each protein in PM1 (Figure 3E). The WPNA of proteome data revealed an association of the RAF-RAS-MAPK pathway with *PD-L1* expression. High protein-level of phospho-c-RAF (p<0.05) and phospho-S6 (p<0.05) were associated with *NF1* loss-of-function (Figure 3C) and resulted in an increase of *PD-L1* expression (Figure 3B, 3C). An additional illustration of associated protein activation downstream of the NF1 pathway is shown in the supplementary Figure 1. Immunostainings of 16 patients (8 Patients with heterozygote *NF1*-deletion 8 patients without *NF1* alteration) of the Freiburg cohort were performed to validate the coherence of MAPK-pathway activation and PD-L1 expression. A significant differences between patients with/without a NF1 loss-of-function. Additionally, p-MAPK14 (p-p38) as a maker of the MAPK-pathway activation was positively correlated (r=0.6, p<0.01) to PD-L1 expression (Figure 4D). P-JNK was also increased in NF-deleted patients, while p-AKT more increased in wild-type patients Figure 4E, 4F.

### Weighted gene co-expression network analysis (WGCNA)

**Figure 5:**
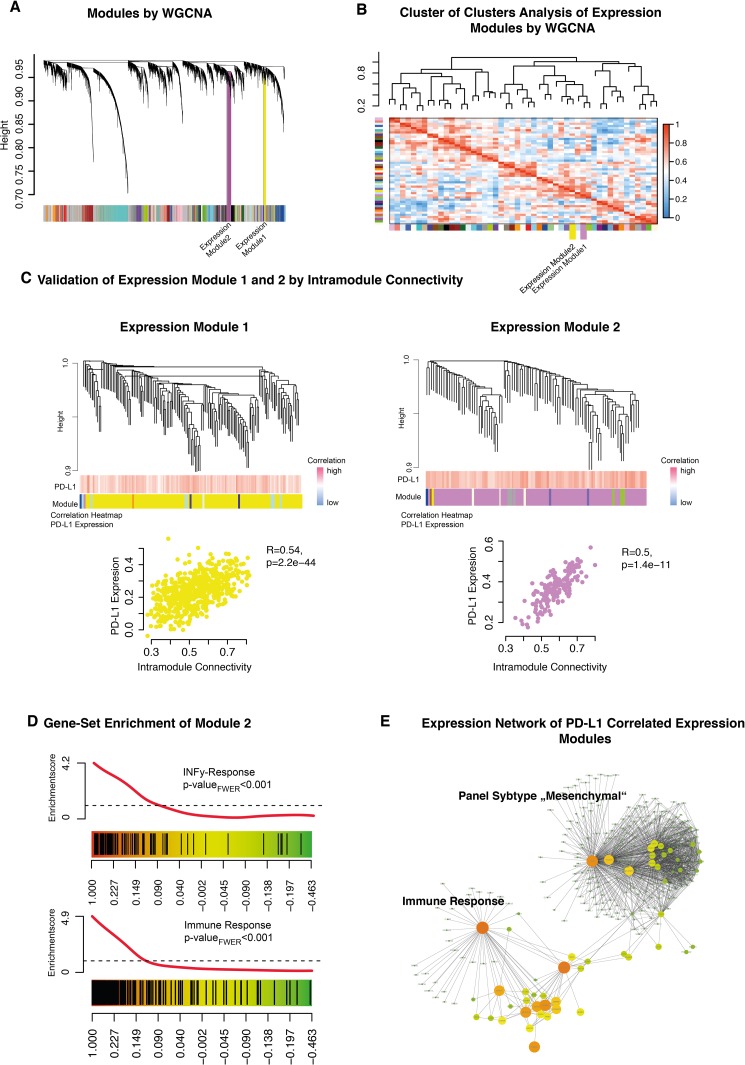
**A**. Weighted gene co-expression network analysis of the whole transcriptome data. **B**. A cluster of clusters analysis presented connected expression modules. Expression module 1 and 2 were contained in one cluster. **C**. Detailed cluster branches of expression module 1 and 2 were illustrated. Corresponding correlation heatmaps of PD-L1 expression and module-contained genes are given in the panel below the branches. Scatterplots of intramodule connectivity (KME) confirmed the strong correlation of PD-L1 expression and the expression modules. **D**. Gene Set Enrichment Analysis of expression module 1 identified INFγ related genes (upper panel) and immune response (bottom panel). **E**. Created network analysis of expression module 1/2 based on intramodule connectivity, derived from WGCNA. Size and colours indicate the intensity of intramodule connectivity.

The WGCNA identified several modules (Figure 5A) that were clustered based on its module eigengene vector (Figure 5B). Two modules were addressed in a cluster together with *PD-L1* expression levels. The correlation of each module and its association to *PD-L1* was validated by direct correlation analysis of *PD-L1* expression and intramodule connectivity.

Expression module 1 (Figure 5C, yellow) was significantly correlated with *PD-L1* expression (R=0.54 p=2.2E-40). Permutation based pre-ranked GSEA was performed and revealed a significant enrichment of mesenchymal subgroup genes (p_FWER_<0.01) (data not shown). Expression module 2 (Figure 5C, violet) was also correlated with *PD-L1* expression (R=0.5 p=1.4E-11). Further GSEA characterised this module as highly associated to immune response and INFγ-activation (p_FWER_<0.01) (Figure 5D). A constructed network based on the intramodule connectivity of module 1 and 2 was presented in Figure 5E. All modules were connected by hub-genes like *CTSO* and *LACTB*, which were part of the immune response related to expression module 2.

## DISCUSSION

### Rationale of the study

Here we present an integrative analysis based on data of the TCGA database and glioblastoma multiforme samples of the Department of Neurosurgery, Medical Center Freiburg. The purpose of this study was to identify potential molecular or genetic alterations in glioblastoma multiforme influencing the *PD-L1* expression. These functional alterations could be used in future diagnostics and help to identify patients who may profit from a PD-L1/PD-1 inhibition.

### Key findings

Firstly, there were significant differences in the expression levels of PD-L1 regarding the different glioma WHO grades, expression subgroups and molecular subgroups (Figure 1B). Lower-grade glioma (LGG, WHO II+III) showed low expression level of *PD-L1* (Figure 1A) compared to glioblastoma multiforme. Secondly, *IDH1/2* wild-type subgroup (Figure 2A) showed similar PD-L1 expression as found in glioblastoma multiforme. This finding supports the recently published hypothesis that *IDH1/2* wild-type subgroup represents typical glioblasoma multiforme-like genetic alterations [22].

Finally, after performing an analysis of frequently occurring genetic alterations in glioblastoma multiforme, *NF1* loss-of-function was significantly associated with strong *PD-L1* expression in both TCGA and Freiburg databases.

### NF1 loss-of-function and RAS-MAPK pathway

The integrative analysis of glioblastoma multiforme proteome data confirmed the correlation between RAS-MAPK pathway and *PD-L1* expression (Figure 3D, 3E). Meanwhile, *NF1* loss-of-function (*NF1* mutation and *NF1*-Deletion) resulted in an activation of MAPK pathway, which affected down-stream targets like MAPK, MEK and JNK (Supplementary Figure 1), thus suggesting a putative impact of *NF1* on the PD-L1 expression. The strong association of MAPK pathway activation in patients with NF1 deletion was validated by immunostainings of the Freiburg cohort (Figure 4). Equally to the WPCNA, a strong correlation between PD-L1 protein-level and p-MAPK14 was shown. Those findings were in line with recently presented results [19] and supported by further analysis identifying a strong correlation of *PD-L1* expression with mesenchymal subgroup genes (Expression module 1, Figure 3E). Additionally, the latter have been recently shown to correlate with *NF1* mutation [21, 24]. As known from the literature, PD-L1 is regulated by the STAT3 transcription factor, which is also a transcriptional master regulator of the mesenchymal subgroup [20, 25]. Parsa et al., 2007 reported an association between PTEN loss and a up-regulation of PD-L1 expression [10]. This association was not found in our analysis which is in line with results reported by Berghoff et al., 2014 [13]. Environmental factors as hypoxia within the up-regulation of *HIF1A* take part in the mesenchymal signature [26]. This expression subgroup was found to be highly connected to immune response and up-regulation of *PD-L1* expression within the tumour [16]. In summary, this study reported a strong coherence between the immune related genes included PD-L1 and the mesenchymal signature of glioblastoma multiforme.

### PD-L1 and immune response

Another interesting finding was the correlation of *PD-L1* expression with the immune response/INFγ activation. This results were in-line with recently published works, showing that pro-inflammatory substrates such as INFγ result in an increased PD-L1 gene expression [5, 27, 28]. GBM are tumours with a large heterogeneity of different cell clones. One major cell population in glioblastoma multiforme was characterized as “immune-cluster” by single-cell RNA sequencing [29]. This immune phenotype was similar to other solid tumours (liver, kidney etc.) [29]. In general, glioblastoma multiforme are tumours that implement a strong immune response within different parts of the tumours and potentially targetable by checkpoint-inhibitors.

### PD-L1 and IDH1/2 mutation

Patients affected by an *IDH1/2* mutation showed a hypermethylated *PD-L1* promoter, which epigenetically silences the *PD-L1* gene expression (Figure 2C, 2D). Of note, recent works showed that treatment with hypomethylating agents in patients with small-cell lung cancer resulted in an increased expression of *PD-L1* [30] underlining the effect of epigenetic regulation on *PD-L1* expression. Epigenetic regulation of PD-L1 is also known for many other cancer types [19].

### Limitation of the study

This study has also some limitations. Firstly, the small sample size and the disregarded heterogeneity could have led to false-positive associations or confounder effects. However, conservative statistical methods with corrections for multiple testing at each level of analysis were applied. Only family-wise error corrected values are reported for the sake of robustness in enrichment analysis.

## MATERIALS AND METHODS

### TCGA data platform

Public available Level 3 TCGA (https://tcga-data.nci.nih.gov/tcga/) data were used for analysis. Data were downloaded at the UCSC Cancer Genome Browser. Expression analysis was performed based on Agilent array data (TCGA GBM G4502A) for high-grade glioma and RNA-seq data (TCGA LGG HiSeqV2 PANCAN) for low-grade glioma. Both datasets were normalized and log2 transformed. For mutation analysis the PANCAN AWG gene-level mutation data set was used for further analysis. Proteomic data were taken from TCGA database as normalized level 3 RPPA data.

### Tissue collection and histology

Tumor tissue was sampled from contrast enhancing regions identified by intraoperative neuronavigation (Cranial Map Neuronavigation Cart 2, Stryker, Freiburg, Germany) during tumor resection. The tissue was snap-frozen in liquid nitrogen immediately after resection and processed for further genetic/metabolic analysis. Tissue samples were fixed using 4% phosphate buffered formaldehyde and paraffin-embedded with standard procedures. H&E staining was performed on 4 μm paraffin sections using standard protocols. Immunohistochemistry was applied using an autostainer (Dako) after heat-induced epitope retrieval in citrate buffer. IDH1 mutation was assessed by immunohistochemistry using an anti-IDH1-R123 antibody (1:20, Dianova).

### Transcriptome analysis

Genome wide expression data of 48 patients (Histology: Glioblastoma multiforme WHO Grade IV) (Department of Neurosurgery, Freiburg, Germany) was used for expression analysis in addition to TCGA data (Clinical data: Supplementary Table 1). RNA was prepared using the RNAeasy kit (Qiagen). An amount of 1.5 μg RNA was obtained for expression arrays analysis. Arrays were performed by human gene ST 2.0 chip (Affymetrix). Raw data were processed, normalized and controlled by R software and the Affymetrix R-package. Data were comparable to Level 3 TCGA data. Molecular subgroups were defined by machine-learning algorithm (random forest) based on TCGA classification (Supplementary Figure 2).

### Genomic data processing

Whole-exome sequencing data (291 patients) of the TCGA database were processed as level III data. Co-mutation analysis was performed in R software and free-available implemented packages. Additional, copy-number variants from 577 patients (Level 3 TCGA data) were analysed. 45 Patients of the Freiburg validation cohort were analysed by Genome-Wide Human SNP Array 6.0 (Affymetrix) and pre-processed by the genome core facility in Freiburg (Department of Hematology, Oncology and Stem Cell Transplantation).

### Weighted gene co-expression analysis

Expression analysis was performed on 585 patients (Array by AgilentG4502A). WGCNA uses the topological overlapping measurement to identify corresponding modules. These modules were analysed by their eigengene correlation to *PD-L1* expression. The WGCNA analysis is a robust tool for integrative network analysis and was used in several recent studies [31–33]. WGCNA established networks were exported to Cytoscape 2.0 [34] for further visualisation. The WGCNA integrated function (exportNetworkToCytoscape) was used to calculate a weighted network. A detailed description of WGCNA was recently reported [35].

### Gene set enrichment analysis (GSEA)

Permutation based gene set enrichment analysis (GSEA) was performed for each module to find specifically enriched biological functions and related pathways [36]. Pre-ranked GSEA were performed with 1000 permutations. P-values were calculated by familywise error rate (FWER), which is a robust method for multiples testing [37]. The Molecular Signatures Database version 5.0 was used including pathways gene sets (C2) (http://www.broadinstitute.org/gsea) as input databases for this analysis. GSEA plots were visualised by limma R-package (barcodeplot function).

### Weighted proteome correlation network analysis

Protein data of 215 patients were extracted from the TCGA database. WPNA was performed for proteome data of the TCGA database and PD-L1 expression. WPCNA was performed with the WCNA-package (R-software) as described above. Modules were built by topological overlapping measurement. The power for network analysis was adapted until scale-free topology was achieved. Derived modules were analysed by their eigengene correlation to *PD-L1* expression level. Additional networks were created based on intermodule connectivity in Cytoscape 2.0 as described above.

### Methylation analysis of PD-L1 promoter

Level 3 methylation data of the TCGA database (155 patients, Methylation 450k) was used for an epigenetic analysis of *PD-L1* promoter. Analysis of the correlation coefficient (Pearson correlation, Fischer's Exact test) between expression and methylation values of all CpG sides of the *PD-L1* promoter was performed as described for MGMT promoter by Bady et al. 2016 [38] (Figure 2C). CpG side “cg19724470” was found as significant negatively correlated to *PD-L1* expression and used for further validation of *PD-L1* promoter methylation (Figure 2D).

### Survival analysis

Overall survival (OS) was available for 45/48 patients (no event for 9 patients, censored). The Kaplan-Meier method was used to provide median point estimates and time-specific rates. The Hazard-Ratio (HR) was calculated by Cox-Regressions tests. Analysis was performed by survival package included in R-Software.

### Immunostaining

The following antibodies were used in immunostaining analyses: phospho-JNK (Phospho-SAPK/JNK (Thr183/Tyr185), Cell signaling, 1:200), p-MAPK14 (p-p38 Antikörper (Thr 180/Tyr 182), Santa Cruz 1:200), PD-L1 (E1LRN, Cell signaling 1:200) and phospho-Akt (Phospho-Akt (Thr 308), Cell signaling, 1:200). Primary antibodies were used at the concentration indicated by the manufacturers. Anti-Mouse and anti-Rabbit Alexa488- or Alexa594-conjugated (Life Technologies) were used as secondary antibodies. Alexa594-conjugated antibodies were used at 1:200 dilution and Alexa488-conjugated antibodies were used at 1:100 dilution. Pictures were acquired using a fluorescent microscope (FL10i, Olympus). Image quantification was performed by ImageJ and analyzed by R-software.

## CONCLUSION

In conclusion, this study reported a comprehensive analysis of *PD-L1* expression in glioblastoma multiforme. *PD-L1* was highly associated with the mesenchymal subgroups and in line with mesenchymal-like genetic alterations like a loss-of-function in *NF1*. Patients with a *NF1* mutations/deletion or RAS-MAPK pathway activation may profit from a targeted therapy with PD-1/PD-L1 inhibition.

## SUPPLEMENTARY MATERIALS FIGURES AND TABLES




