# Supplementary material for: Comprehensive analysis of PD-L1 expression in glioblastoma multiforme
Source: Oncotarget. 2017 Feb 2;8(26):42214–25. doi: 10.18632/oncotarget.15031 (PMC5522061; doi:10.18632/oncotarget.15031)
Supplement: Supplementary file 1 [file oncotarget-08-42214-s001.pdf]

Comprehensive analysis of PD-L1 expression in glioblastoma multiforme

SUPPLEMENTARY MATERIALS

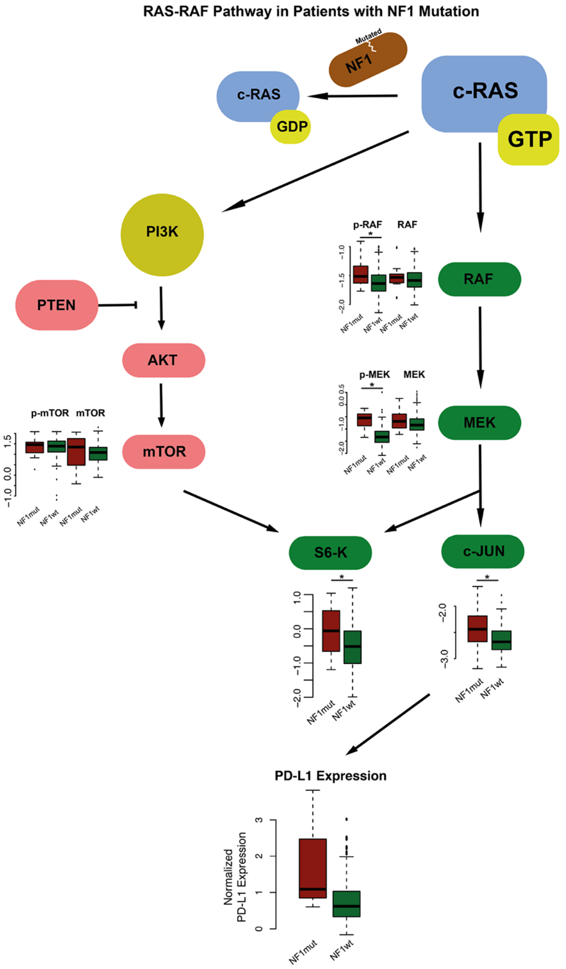

Supplementary Figure 1: Map of the MAPK-pathway in NF1-mutated patients.

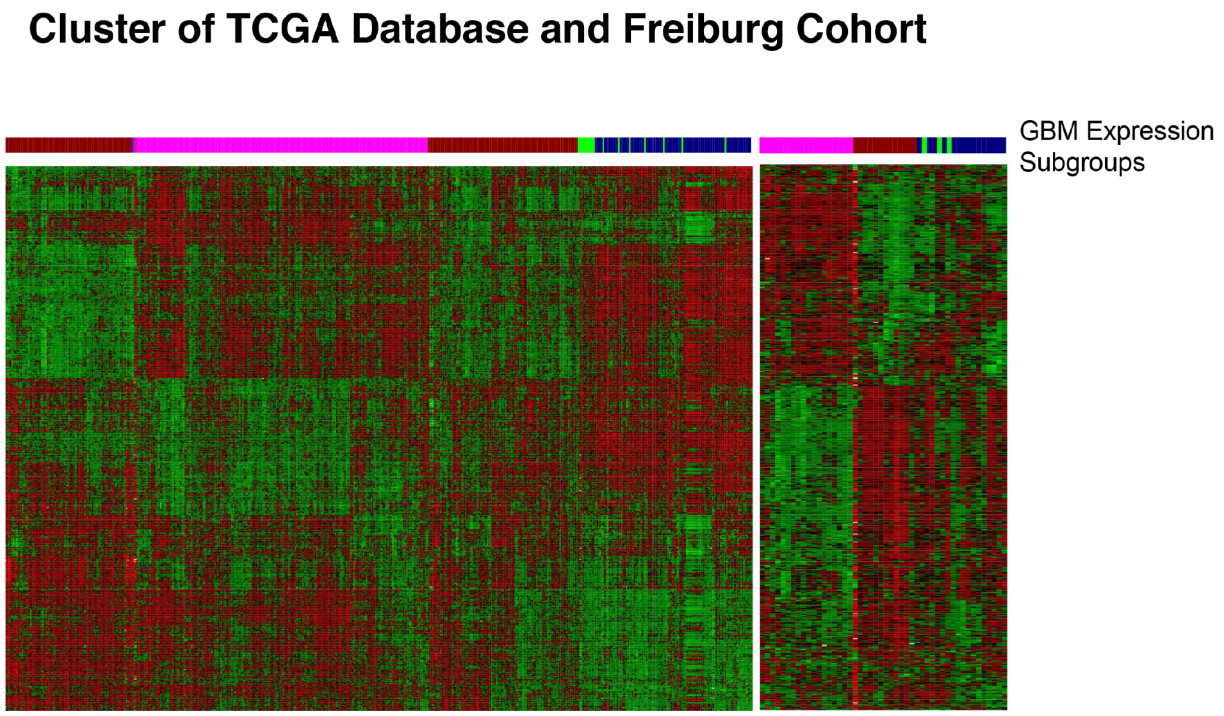

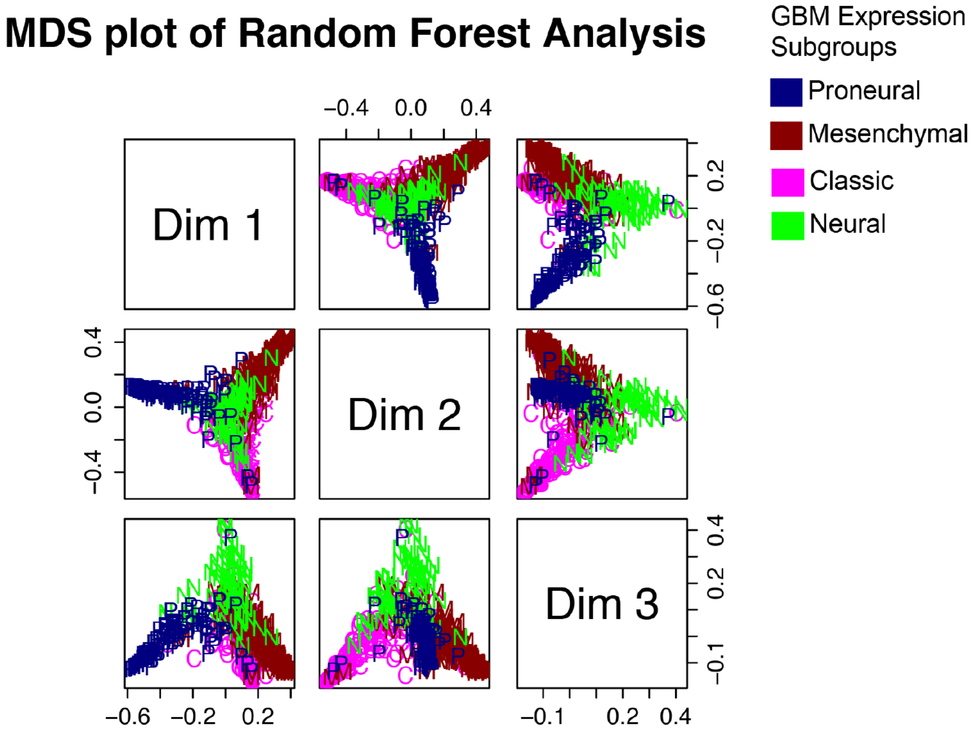

Supplementary Figure 2: Classification of freiburg cohort.

Supplementary Table 1: Clinical characteristics of Freiburg cohort

See Supplementary File 1
