# Supplementary material for: Comprehensive analysis of PD-L1 expression in glioblastoma multiforme
Source: Oncotarget. 2017 Feb 2;8(26):42214–25. doi: 10.18632/oncotarget.15031 (PMC5522061; doi:10.18632/oncotarget.15031)
Supplement: Supplementary file 2 [file oncotarget-08-42214-s002.docx]

Supplementary Table 1: Clinical Characteristics of Freiburg Cohort

| **Patient** | **OS** | **Sampling** | **Geschlecht** | **Diagnosis** | **Geb. Datum** |
| --- | --- | --- | --- | --- | --- |
| **Pat_1** | 697 | 22.12.11 | m | GBM | 25.08.71 |
| **Pat_2** | 489 | 23.07.12 | w | GBM | 30.06.50 |
| **Pat_3** | 214 | 19.03.14 | m | GBM | 14.11.51 |
| **Pat_4** | 345 | 09.11.12 | m | GBM | 07.12.40 |
| **Pat_5** | 1168 | 25.04.13 | m | GBM | 18.10.36 |
| **Pat_6** | 46 | 21.09.12 | w | GBM | 21.06.30 |
| **Pat_7** | 1221 | 07.09.12 | w | GBM | 25.06.41 |
| **Pat_8** | 420 | 07.04.14 | m | GBM | 28.02.73 |
| **Pat_9** | 50 | 20.03.12 | w | GBM | 10.05.74 |
| **Pat_10** | 391 | 14.11.13 | m | GBM | 07.04.49 |
| **Pat_11** | 1405 | 30.05.11 | w | GBM | 12.06.66 |
| **Pat_12** | 117 | 02.05.13 | w | GBM | 14.02.65 |
| **Pat_13** | 552 | 22.07.10 | w | GBM | 19.02.45 |
| **Pat_14** | NA | 17.09.10 | w | GBM | 18.03.54 |
| **Pat_15** | 642 | 10.04.12 | m | GBM | 09.07.62 |
| **Pat_16** | 597 | 25.05.11 | m | GBM | 02.05.43 |
| **Pat_17** | 417 | 15.07.10 | m | GBM | 04.08.75 |
| **Pat_18** | 827 | 10.03.14 | w | GBM | 05.01.55 |
| **Pat_19** | 757 | 25.03.10 | w | GBM | 19.04.65 |
| **Pat_20** | 572 | 30.12.10 | m | GBM | 04.06.86 |
| **Pat_21** | 835 | 26.01.11 | m | GBM | 01.03.65 |
| **Pat_22** | 621 | 29.03.11 | w | GBM | 01.05.38 |
| **Pat_23** | 72 | 19.11.10 | m | GBM | 25.08.41 |
| **Pat_24** | 156 | 07.10.10 | m | GBM | 16.12.51 |
| **Pat_25** | 494 | 12.08.11 | m | GBM | 24.02.45 |
| **Pat_26** | NA | 16.06.11 | m | GBM | 13.03.59 |
| **Pat_27** | 172 | 21.10.10 | m | GBM | 08.10.69 |
| **Pat_28** | 1461 | 04.10.10 | w | GBM | 14.10.46 |
| **Pat_29** | 668 | 20.05.11 | m | GBM | 27.05.57 |
| **Pat_30** | 572 | 08.10.10 | m | GBM | 29.09.46 |
| **Pat_31** | 125 | 13.09.10 | m | GBM | 31.03.40 |
| **Pat_32** | NA | 02.09.10 | m | GBM | 28.12.44 |
| **Pat_33** | 486 | 29.03.11 | w | GBM | 13.06.45 |
| **Pat_34** | 841 | 25.07.11 | w | GBM | 17.10.38 |
| **Pat_35** | NA | 29.11.10 | w | GBM | 05.04.69 |
| **Pat_36** | 190 | 28.06.10 | m | GBM | 30.05.43 |
| **Pat_37** | NA | 11.01.11 | m | GBM | 04.06.97 |
| **Pat_38** | 271 | 19.06.12 | w | GBM | 05.04.31 |
| **Pat_39** | 1383 | 11.10.12 | w | GBM | 28.07.52 |
| **Pat_40** | 297 | 10.03.15 | w | GBM | 15.04.69 |
| **Pat_41** | 733 | 22.03.13 | m | GBM | 20.01.50 |
| **Pat_42** | 112 | 20.12.12 | m | GBM | 09.12.39 |
| **Pat_43** | NA | 08.01.13 | w | GBM | 06.12.78 |
| **Pat_44** | 323 | 12.02.15 | w | GBM | 25.06.65 |
| **Pat_45** | 451 | 23.09.14 | w | GBM | 02.12.64 |
| **Pat_46** | 279 | 09.11.12 | w | GBM | 19.07.47 |
| **Pat_47** | 122 | 19.03.12 | m | GBM | 01.05.38 |
| **Pat_48** | 137 | 17.09.12 | m | GBM | 18.06.46 |
